# Prime immunization with rotavirus VLP 2/6 followed by boosting with an adenovirus expressing VP6 induces protective immunization against rotavirus in mice

**DOI:** 10.1186/1743-422X-8-3

**Published:** 2011-01-05

**Authors:** Hongli Zhou, Li Guo, Min Wang, Jianguo Qu, Zhendong Zhao, Jianwei Wang, Tao Hung

**Affiliations:** 1 National Institute for Viral Disease Control and Prevention, Chinese Center for Disease Control and Prevention, Beijing 100052, PR China; 2 State Key Laboratory for Molecular Virology and Genetic Engineering, Institute of Pathogen Biology, Chinese Academy Medical Sciences & Peking Union Medical College, Dong Dan San Tiao, Beijing 100730, PR China

## Abstract

**Background:**

Rotavirus (RV) is the main cause of severe gastroenteritis in children. An effective vaccination regime against RV can substantially reduce morbidity and mortality. Previous studies have demonstrated the efficacy of virus-like particles formed by RV VP2 and VP6 (VLP2/6), as well as that of recombinant adenovirus expressing RV VP6 (rAd), in eliciting protective immunities against RV. However, the efficacy of such prime-boost strategy, which incorporates VLP and rAd in inducing protective immunities against RV, has not been addressed. We assessed the immune effects of different regimens in mice, including rAd prime-VLP2/6 boost (rAd+VLP), VLP2/6 prime-rAd boost (VLP+rAd), rAd alone, and VLP alone.

**Results:**

Mice immunized with the VLP+rAd regimen elicit stronger humoral, mucosal, and cellular immune responses than those immunized with other regimens. RV challenging experiments showed that the highest reduction (92.9%) in viral shedding was achieved in the VLP+rAd group when compared with rAd+VLP (25%), VLP alone (75%), or rAd alone (40%) treatment groups. The reduction in RV shedding in mice correlated with fecal IgG (r = 0.95773, *P *= 0.04227) and IgA (r = 0.96137, *P *= 0.038663).

**Conclusions:**

A VLP2/6 prime-rAd boost regimen is effective in conferring immunoprotection against RV challenge in mice. This finding may lay the groundwork for an alternative strategy in novel RV vaccine development.

## Background

Rotavirus (RV) infection is the most common cause of severe gastroenteritis in children. RV-induced gastroenteritis is responsible for over 600,000 deaths of children every year; 85% of these deaths occur in developing countries where nearly two million children are hospitalized annually due to RV infection [1,2].

The US Food and Drug Administration (FDA) licensed the first RV vaccine (Rotashield™) in 1998. However, this vaccine was withdrawn only one year later due to a common side effect, intussusception [3]. In recent years, two more live RV vaccines, Rotarix™ (an attenuated human RV strain developed by GlaxoSmithKline) and Rotateq™ (a pentavalent human-bovine reassortant developed by Merck) were licensed in several countries [4-6]. Yet the protective mechanisms of these RV vaccines have not been fully understood [7].

Previous studies have shown that RV VP6 can interact with a large fraction of human naive B cells [8] and that the immunization using VP6 protein or DNA can induce protective immunities in mice, gnotobiotic pigs, and other animal models [9-14]. It has also been shown that the double layered virus-like particles (VLPs) formed by VP2 and VP6 (VLP2/6) of RV [15], together with mucosal adjuvant, are able to induce protective immunities [16-19]. These studies strongly suggest that VP6 plays a key role in RV protective immunity.

Recombinant adenoviruses (rAds) have been widely used in the development of viral vaccines due to their safety and effectiveness in gene transfer and expression [20-24]. Administration of rAd expressing human RV VP6 orally or intranasally stimulates effective specific humoral, mucosal, and cellular immune responses and confers protection against RV infection in mice [25]. Studies have also shown that combining rAds with DNA or protein in prime-boost strategies effectively enhance the immune response against target antigens. Such methods have been applied to the development of vaccines against HIV and many other viruses [26-29].

In the present study, we investigated the efficacy of prime-boost regimens in eliciting specific protective immunities against RV infection in mice. We found that mice immunized with VLP2/6 prime-rAd boost regimen elicit stronger humoral, mucosal and cellular immune responses and confer stronger protection against RV challenge than those immunized with other regimens. Our data suggest the use of a VLP prime-rAd boost strategy for the development effective RV vaccines.

## Results

### Humoral immune responses

**Figure 1:**
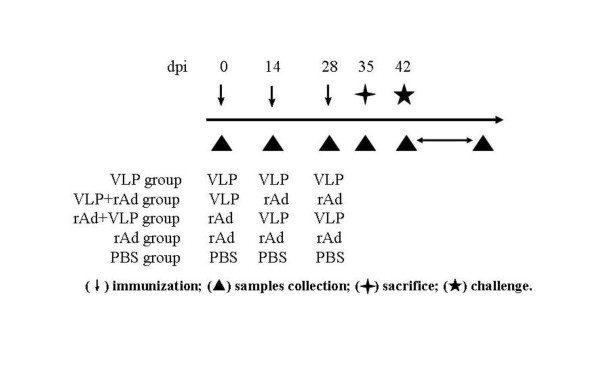
**Schemes for animal experiments and sample collection**. BALB/c mice were randomized into five groups and were immunized and sampled as described in the *Materials and Methods *section. Mice were sacrificed at 35 days post-inoculation (dpi) and the cellular immune responses were determined. At dpi 42, the remaining mice were challenged with the murine RV EDIM strain, and stool samples were collected daily from dpi 42 to dpi 53.

**Figure 2:**
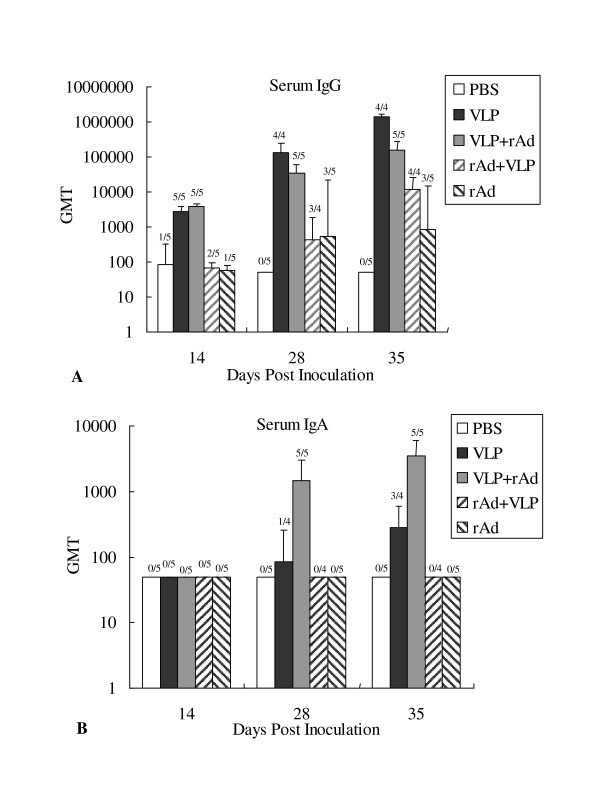
**Serum RV VP6 specific antibody response following immunization**. Serum samples were collected from each mouse at 14, 28, and 35 days post-inoculation (dpi). Serum RV specific IgG (A) and IgA (B) antibodies from individual mice were determined by ELISA and used to calculate the GMTs for each group of mice. Days post inoculation are shown on the X-axis. Error bars represent standard errors of the means. Above each column is the number of responders over the total number of mice tested.

To asses the effectiveness of different vaccination regimens in eliciting specific humoral responses in mice (Figure 1), serum IgG and IgA targeted to RV were analyzed by indirect ELISAs. We found that after the first immunization (14 days post-inoculation), anti-VP6 IgG were present in all mice subjected to VLP+rAd and VLP treatment. Moreover, after the third immunization (35 dpi), the anti-VP6 IgG antibody levels of the VLP+rAd group (GMT = 160948) and the VLP group (GMT = 1377449) were significantly higher than those of the other two groups [VLP+rAd group vs. rAd+VLP group (GMT = 11771), *P *= 0.02033; VLP +rAd group vs. rAd group (GMT = 852), *P *= 0.00747; VLP group vs. rAd+VLP group, *P *= 0.00126; VLP group vs. rAd group, *P *= 0.00246]. Anti-VP6 IgG were present in all of the mice in the rAd+VLP group until after the third immunization. In the rAd group seroconversion was observed in only 3 out of 5 mice (Figure 2A).

Anti-VP6 IgA were not detected at dpi14 in any groups. However, these antibodies appeared at dpi 28 and dpi 35 only in mice immunized with VLP+rAd and VLP (Figure 2B). The IgA level of the VLP +rAd group was the highest, and at dpi 28, all mice in this group were positive for anti-VP6 IgA. At dpi 35, the serum IgA of the VLP+rAd group (GMT = 3482) was significantly higher than that of the VLP group (GMT = 283, *P *= 0.00425). In the VLP group, only 3/4 of the mice showed that IgA were positive at dpi 35. The serum anti-VP6 IgA in the rAd+VLP group and rAd alone group remained negative in the duration of the study (Figure 2B).

These results demonstrate that, among the four strategies tested, the VLP2/6 prime-rAdVP6 boost strategy was the most effective in inducing the humoral immune response against RV VP6 in mice.

### Mucosal immune responses

**Figure 3:**
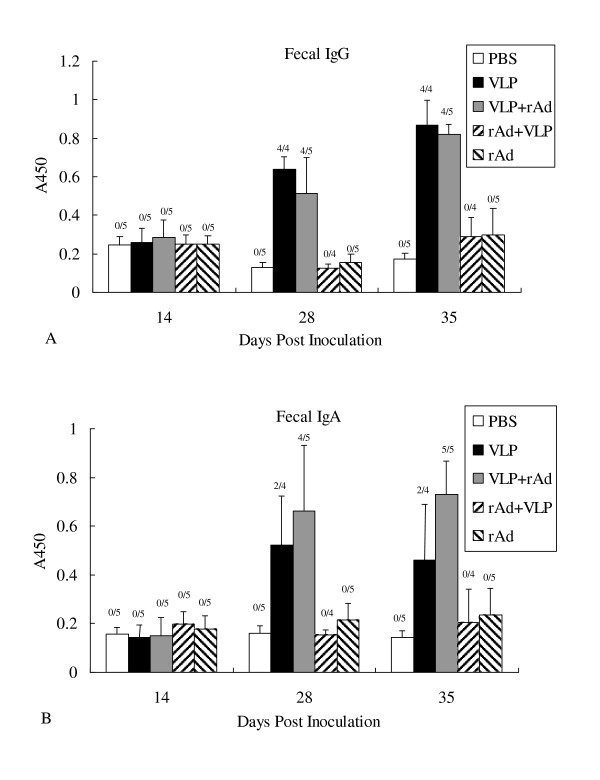
**Fecal RV VP6 specific antibody response following immunization**. Fecal samples were collected from each mouse at 14, 28, and 35 days post-inoculation (dpi). Levels of specific IgG (A) and IgA (B) antibodies in the feces were examined by indirect ELISAs. Days post inoculation are shown on the X-axis. Error bars show the standard errors of the mean. Above each column is the number of responders over the total number of mice tested.

We assessed the ability of various immunization regimen in inducing specific mucosal antibody responses by determining the level of RV VP6 specific IgG (Figure 3A) and IgA (Figure 3B) in fecal matter. Fecal suspensions were measured after the third immunizations. Our results showed that at dpi 14, IgA and IgG were both negative in all experimental and control groups. After the second immunization, the A450 of IgA in the VLP+rAd group and in the VLP group increased to 0.663 ± 0.267 and 0.524 ± 0.200, with an increasing of IgG to 0.513 ± 0.184 and 0.639 ± 0.064, respectively, at A450. At dpi 35, the A450 of IgA in the VLP+rAd group and in the VLP group increased to 0.73 ± 0.14 and 0.46 ± 0.23, while the A450 of IgG increased to 0.82 ± 0.05 and 0.87 ± 0.13, respectively. But there was no significant differences between the fecal IgA (*P *= 0.17412) and IgG (*P *= 0.34917) level of the two groups. Notably, the anti-VP6 IgA and IgG in the PBS, rAd+VLP, and rAd groups were negative after each inoculation.

In the VLP+rAd group, 4 of 5 mice tested were positive for anti-VP6 IgA at dpi 28 and all mice were positive at dpi 35. This is in contrast to the VLP treated group for which only 2 of 4 mice tested IgA positive at dpi 35. Furthermore, all the VLP treated mice tested positive for the presence of anti-VP6 IgG in fecal matter at dpi 28, whereas 4 out of 5 mice in the VLP+rAd group were positive at dpi 28 and dpi 35. These results indicate that the VLP+rAd regimen is more effective than the other regimens tested in eliciting mucosal immune response.

### Cellular immune responses

**Figure 4:**
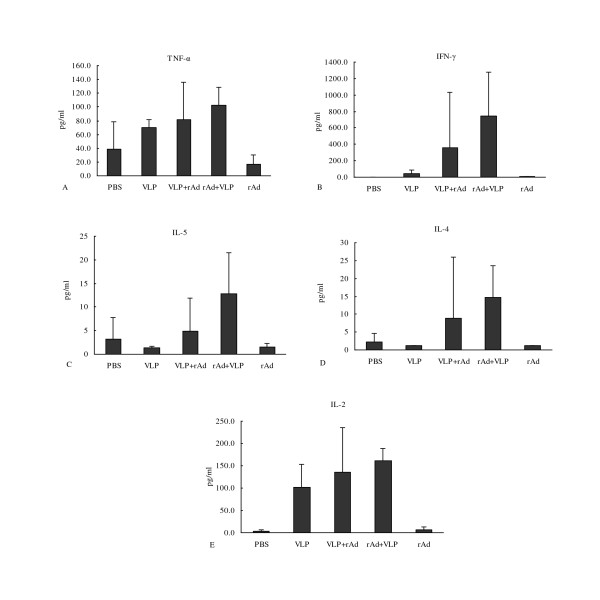
**Cytokine production by splenocytes from immunized mice**. Mice were sacrificed seven days after three immunizations. The splenocytes were isolated and stimulated with RV VP6 peptide. The concentrations of TNF-α (A), IFN-γ (B), IL-5 (C), IL-4 (D) and IL-2 (E) in the culture supernatant were measured. Error bars represent standard errors of the mean.

Secreted cytokines (TNF-α, IFN-γ, IL-5, IL-4 and IL-2) were analyzed by CBA technology to profile the cellular immune responses to the different vaccination regimens (Figure 4). We found that the levels of both Th1 cytokines (TNF-α, IFN-γ, and IL-2) and Th2 cytokines (IL-4 and IL-5) increased following all immunization schemes. Although we did not detect statistical differences in the level of these specific cytokines, mice in the VLP+rAd and the rAd+VLP group exhibited higher cytokine levels overall. The TNF, IL-4, and IL-5 secretion in the VLP group (TNF 70.5 pg/ml; IFN-γ 40.3 pg/ml; IL-2 101.0 pg/ml; IL-4 1.2 pg/ml; IL-5 1.3 pg/ml) were nearly the same as that of the PBS group (TNF 39.1 pg/ml; IFN-γ 1.2 pg/ml; IL-2 2.6 pg/ml; IL-4 2.3 pg/ml; IL-5 3.1 pg/ml). Only IFN-γ and IL-2 levels were higher than those of the PBS group. All cytokines in the rAd group (TNF 16.3 pg/ml; IFN-γ 4.5 pg/ml; IL-2 6.2 pg/ml; IL-4 1.2 pg/ml; IL-5 1.4 pg/ml) were essentially the same as those in the PBS group.

### Protective efficacy against RV challenge

**Figure 5:**
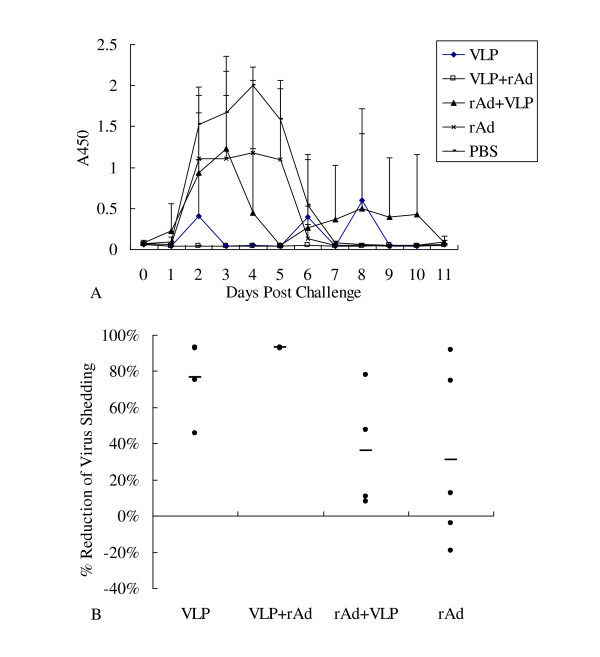
**Protection from RV shedding in mice following immunization**. Five mice from each group were challenged with the murine RV EDIM strain at dpi 42, and stool samples were collected daily from dpi 42 to dpi 53. The presence of RV antigen in fecal samples (A) was determined by a sandwich-ELISA. Reduction in shedding (B) was calculated for each animal by comparing the area under the curve for each individual animal to the mean of the areas under the curves of the control group.

To determine the protection conferred by VLP2/6 prime-rAdVP6 boost, rAdVP6 prime-VLP2/6 boost, as well as VLP2/6 and rAdVP6 alone, five mice from each group were challenged with 10×DD50 of murine RV EDIM at dpi 42. Viral shedding curves (Figure 5A) indicated that the mice in the PBS group shed virus as early as 2 days after challenge. The viral shedding in each experimental group decreased to various degrees after challenge. Reduction in shedding (Figure 5B) of the VLP+rAd group was the highest (92.9%), with more than 50% of reduction in each mouse. Reductions in shedding of the VLP group, the rAd+VLP group, and the rAd group were 76.7%, 36.1%, and 31.1%, respectively. These numbers were lower than those of the VLP+rAd group, and varied largely among individuals in each group. Our results suggest that the VLP2/6 prime-rAdVP6 boost regimen is more effective than other regimen in conferring immunoprotection against RV challenge in mice.

## Discussion

In the present study, we compared the effectiveness of VLP prime-rAd boost and rAd prime-VLP boost regimens in eliciting anti-RV protective immunities. Our results demonstrate that the VLP2/6 prime-rAdVP6 boost regimen is more effective in stimulating VP6 specific immunities and conferred a higher protection than the other regimens tested.

We administered mice with VLP2/6 via an intranasal route to elicit vigorous mucosal immunity [18,30,31]. In contrast, rAdVP6 was administered via an oral route to ensure the safety of using adenovirus as a component of a vaccine [32]. Studies have shown that immune response elicited by oral rAd administration are poor even in large doses [25,33]. We used a relatively small dosage of adenovirus in each immunization (10^6^ifu/dosage, approximately 1/100-1/10 of the documented doses[34]) and found that the immune responses induced by rAd alone were similar to those of the PBS group, indicating that rAd alone was unable to protect the mice against RV challenge.

Repeated immunization of VLP2/6 can effectively induce humoral and mucosal immunity, but the induction of cellular immunity was not as effective as the prime-boost regimens (VLP+rAd or rAd+VLP). After the RV challenge, the mice immunized with VLP alone still showed obvious virus shedding, with a large variation of shedding amount between individuals within the group. In contrast, the VLP+rAd group not only elicited high level humoral, mucosal, and cellular immunities, but also protected against RV challenge and effectively reduced the amount of virus shedding. After VLP priming, boosting twice with rAd at a small dosage was an effective and economical immunization scheme. Our results indicate that a prime-boost regimen may have synergetic immune effects.

In our study, the mice immunized with the VLP+rAd regimen elicited stronger humoral, mucosal, and cellular immune responses than those immunized with other regimens. The reasons for this disparity are unclear. One possible explanation may be the difference in inducing innate immunity between rAd and VLP, which leads to a difference in type and strength of the adaptive immune responses [29]. VLP and rAd are recognized by different pattern recognition receptors, such as Toll-like receptors [35,36], which may lead to differences in cytokine activation. The sequence of prime-boost immunization may also affect the cytokine milieu. This milieu may determine the final direction, strength, and breadth of various adaptive immunities, including the balance between Th1 and Th2 immune responses through different mechanisms [37]. However, these mechanisms cannot be unravelled by our data alone. A systems biology approach to analyze the markers of the immune responses by different prime-boost regimens may be needed [38].

Although the molecular mechanisms regulating immunoprotection against RV are still unclear and the immunological indicators that can accurately reflect protection against RV infection remain to be established, mucosal immunity appears to be important in anti-RV protective immunities [11,13,30,39-44]. Our correlation analysis between various immune indicators and reduction in RV shedding in mice indicate that a reduction in shedding depends on the levels of fecal IgG antibody (r = 0.95773, *P *= 0.04227) and IgA antibody (r = 0.96137, *P *= 0.038663) (see Table 1). This finding suggests that protection against RV is correlated with local intestinal mucosal immunities. The observation is consistent with the finding that immunities evoked by VP6 are mainly present in intestines [45].

**Table 1:** Correlation analysis between all measurement indicators and reduction in rotavirus shedding in mice

Indicators	r	*P *value
Serum IgA	0.94839	0.051611
Serum IgG	0.84071	0.15929
Fecal IgA	0.96137	0.038633
Fecal IgG	0.95773	0.04227
TNF-α	0.37996	0.62004
IFN-γ	-0.0793	0.92072
IL-5	-0.2375	0.76253
IL-4	0.01328	0.98672
IL-2	0.48413	0.515787

Several studies have suggested that cellular immunity plays an important role in the clearance of RV infection [14,46-48]. However, although the rAd+VLP regimen induced a strong T cell response, we did not observe a correlation between this reaction and protective efficacy. Future studies with multiple methods and epitopes may be necessary to determine the cellular immune responses more precisely and to assess their significance in anti-RV immunities.

## Conclusions

Our study has shown that a VLP2/6 prime-rAdVP6 boost regimen elicits protective immunities from RV infection and is a superior regimen to those of VLP2/6 prime-rAdVP6 boost, VLP2/6 alone, or rAdVP6 alone. Thus, the VLP2/6 prime-rAdVP6 boost regimen may provide an alternative strategy for novel RV vaccine development.

## Methods

### Preparation of recombinant adenovirus and VLP2/6

The recombinant replication defective adenovirus serotype 5 (Ad5) expressing RV VP6, termed rAdVP6, was generated with the AdEasy system (Stratagen, Cedar Creek, TX) following the manufacturer's instructions. Expression of VP6 was confirmed by Western blot analysis using an antibody against RV (Biodesign, Cat: B65110G). The virus was titered with an Adeno-X Rapid Titer Kit (BD Biosciences Clontech, Mountain View, CA) and stored at -70°C prior to use.

VLP2/6 was produced by expression of RV VP2 and VP6 simultaneously in *Spodoptera frugiperda *(Sf9) cells through recombinant baculovirus. The recombinant baculovirus was generated by the Bac-to-Bac^® ^Baculovirus Expression System (Invitrogen, Carlsbad, CA) according to the manufacturer's protocol. RV VLP2/6 was purified by ultracentrifugation as described previously [49,50]. Briefly, the supernatants of Sf9 cells infected by the recombinant baculovirus were collected at day 5 post infection and cellular debris was removed by centrifugation (20 min at 10,000 rpm). VLP2/6 was precipitated with PEG6000 (final concentration, 6%) from the clarified supernatant. Precipitated pellets were sonicated briefly followed by ultracentrifugation at 35,000 rpm for 3 hours through a 40% sucrose cushion. The presence of the purified VLP2/6 was confirmed by Western blot using an anti-RV antibody. Concentrated VLP2/6 were verified by electron microscopy. The concentration of purified VLP2/6 protein was determined using the BCA Protein Assay Reagent Kit (Pierce, Rockford, IL), and proteins were stored at -70°C prior to use.

### Prime-boost regimens and animal experiments

Six- to eight-week old female BALB/c mice were obtained from the Institute of Laboratory Animal Science, Chinese Academy of Medical Sciences, and maintained in Animal Biosafety Level-2 facilities. Mice were confirmed to be RV and Ad5 antibody-free by ELISA prior to immunization and were randomized into one of the five treatment groups as shown in Figure 1. For the VLP group, mice were intranasally (i.n.) inoculated with 10 μg RV VLP2/6 at days 0, 14, and 28, respectively. For the VLP+rAd group, mice were i.n. primed with 10 μg RV VLP2/6 at day 0, followed by twice oral boosting of 1 × 10^6 ^ifu (infectious units) rAdVP6 (in 0.1 ml each dose) at days 14 and 28, respectively. For the rAd+VLP group, mice were orally primed with 1 × 10^6 ^ifu of rAdVP6 (in 0.1 ml each dose) at day 0, followed by twice i.n. boosting with 10 μg RV VLP2/6 at days 14 and 28. For the rAd group, mice were orally inoculated with 1 × 10^6 ^ifu rAdVP6 (in 0.1 ml each dose) at days 0, 14, and 28. In all the cases of VLP2/6 administration, 10 μg of CpG ODN 1826 (5' > TCC ATG ACG TTC CTG ACG TT < 3', synthesized by Shanghai Sangon Biological Engineering Technology & Services Co., Ltd., Shanghai, China), and 1 μg poly I:C (Sigma, St. Louis, MO) per dose were used as adjuvant. Control mice (PBS group) received intranasal immunization of 0.1 ml PBS at days 0, 14, and 28.

At 0, 14, 28, and 35 days post-inoculation (dpi), serum and stool samples were collected from each mouse before each immunization. Sera were stored at -20°C until analysis. Five mice from each group were euthanized at dpi 35 and splenocytes were isolated for the cytokine measurements. The remaining five mice from each group were challenged with a 10 × 50% diarrhea-inducing dose (DD50) of murine EDIM RV at 42 dpi and stool samples were collected daily from dpi 42 to 53. Feces were weighed and resuspended in PBS (pH 7.2; 1:10, wt/vol). Debris was removed by centrifugation and supernatants were stored at -20°C until analysis.

### Measurement of RV-specific antibodies by ELISA

Ninety-six-well polystyrene microtiter plates (Costar, Bethesda, MD) were coated overnight at 4°C with 0.1 μg/well VP6 antigen diluted in carbonate buffer after optimization of the experiments. Wells were washed three times with 0.05% (vol/vol) Tween 20 in PBS (PBS-T) and blocked with 200 μl of 1% BSA (Sigma, St. Louis, MO) in PBS (PBS-BSA) for 2 hours at 37°C. After washing, 100 μl/well of serum or stool homogenates diluted in PBS-BSA were added, and plates were incubated for 1 hour at 37°C to prevent non-specific binding. Subsequently, plates were washed and incubated for 1 hour at 37°C with 100 μl/well of horseradish peroxidase (HRP)-labeled anti-mouse immunoglobulin G (IgG) or IgA (Sigma, St. Louis, MO) at a dilution of 1:5000 in PBS-BSA. Color was developed by adding 100 μl/well of Sure Blue TMB (Sigma, St. Louis, MO) peroxidase substrate, and absorbance was read at 450 nm (A450) using an BioRad 550 ELISA plate reader (BioRad, Hercules, CA). Serums were two-fold serially diluted to determine antibody titers.

### Detection of RV antigen in stools

The presence of RV antigen in fecal samples was determined by a sandwich-ELISA using a Rotavirus Assay Kit (Lanzhou Institute of Biological Products, Lanzhou, China) according to the manufacturer's protocol. Individual stool samples were tested--10% (wt/vol)--and specimens' A450 was determined using an ELISA plate reader (BioRad 550, Hercules, CA). Viral shedding curves for each animal were plotted, and the areas under the curves for each animal were calculated. Reduction in shedding was calculated for each immunized animal by comparing the area under the curve to the mean of the areas under the curves of the control group. Reduction in shedding was then calculated for each vaccination group by determining the mean reduction of each vaccinating group. A >50% reduction in virus shedding for an individual animal or for a group was considered significant protection from virus challenge.

### Multiple-cytokine assays

Freshly isolated murine splenocytes were cultured on 96-well round-bottom tissue culture plates at 5 × 10^5 ^cells/well in complete RPMI 1640 medium (Invitrogen, Carlsbad, CA). Cells were stimulated with VP6 peptide [9,51] (RLSFQLMRPPNMTP, synthesized by the Chinese Academy of Military Medical Sciences) for 48 hours. Supernatants were collected and IL-2, IL-4, IL-5, TNF-α, and IFN-γ secretion were quantified using the Mouse Th1/Th2 Cytokine Cytometric Array Bead (CBA) Kit (BD PharMingen, San Diego, CA) according to the manufacturer's protocol. The IL-2, IL-4, IL-5, TNF-α, and IFN-γ secretion were detected with FACSCalibur^® ^Flow Cytometer (BD Biosciences, San Jose, CA) using two-color detection and analyzed using CBA software (BD PharMingen).

### Statistical analysis

Antibody titers were log10-transformed and expressed as geometric mean titers (GMTs). When RV-specific antibodies were not detected, a value of 50 (one-half the lowest detectable level) was assigned to that sample, and used in the calculation of the mean and standard error. When the value of the sample was two times that of the background, it was considered positive. Differences between groups were compared by Student's t-test. Correlation analysis was performed by Pearson correlation. All tests were two-tailed, and a P value of <0.05 was considered significant.

## Competing interests

The authors declare that they have no competing interests.

## Authors' contributions

HZ, LG and MW: constructed and characterized VLP2/6 and rAdVP6, immunized mice and evaluated the immune response. JQ: characterized VLP2/6 with electron microscopy. HZ and ZZ, JW: wrote the manuscript. ZZ, JW and TH: participated in the interpretation of data and critically revised the manuscript. All authors read and approved the final manuscript.
